# Synaptotagmin 1 Is Involved in Neuropathic Pain and Electroacupuncture-Mediated Analgesic Effect

**DOI:** 10.3390/ijms21030968

**Published:** 2020-01-31

**Authors:** Juan Wan, Sha Nan, Jingjing Liu, Mingxing Ding, Hongmei Zhu, Chuanguang Suo, Zhuole Wang, Manli Hu, Dehai Wang, Yi Ding

**Affiliations:** College of Veterinary Medicine, Huazhong Agricultural University, Wuhan 430070, China; wanjuanyimi@163.com (J.W.); yunheng3@163.com (S.N.); Ljj779009242@163.com (J.L.); dmx@mail.hzau.edu.cn (M.D.); han.dong.1988@163.com (H.Z.); SCGwonderful@163.com (C.S.); wangzhuole@foxmail.com (Z.W.); humanli0727@163.com (M.H.); wangdehai@mail.hzau.edu.cn (D.W.)

**Keywords:** Synaptotagmin 1, electroacupuncture, neuropathic pain, allodynia, hyperalgesia

## Abstract

Numerous studies have verified that electroacupuncture (EA) can relieve neuropathic pain through a variety of mechanisms. Synaptotagmin 1 (Syt-1), a synaptic vesicle protein for regulating exocytosis of neurotransmitters, was found to be affected by EA stimulation. However, the roles of Syt-1 in neuropathic pain and EA-induced analgesic effect remain unclear. Here, the effect of Syt-1 on nociception was assessed through an antibody blockade, siRNA silencing, and lentivirus-mediated overexpression of spinal Syt-1 in rats with spared nerve injury (SNI). EA was used for stimulating bilateral “Sanjinjiao” and “Zusanli” acupoints of the SNI rats to evaluate its effect on nociceptive thresholds and spinal Syt-1 expression. The mechanically and thermally nociceptive behaviors were assessed with paw withdrawal threshold (PWT) and paw withdrawal latency (PWL) at different temperatures, respectively, at day 0, 7, 8, 14, and 20. Syt-1 mRNA and protein levels were determined with qRT-PCR and Western blot, respectively, and its distribution was observed with the immunohistochemistry method. The results demonstrated Syt-1 antibody blockade and siRNA silencing increased ipsilateral PWTs and PWLs of SNI rats, while Syt-1 overexpression decreased ipsilateral PWTs and PWLs of rats. EA significantly attenuated nociceptive behaviors and down-regulated spinal Syt-1 protein levels (especially in laminae I-II), which were reversed by Syt-1 overexpression. Our findings firstly indicate that Syt-1 is involved in the development of neuropathic pain and that EA attenuates neuropathic pain, probably through suppressing Syt-1 protein expression in the spinal cord.

## 1. Introduction

Injury to central or peripheral nerves or illnesses often leads to neuropathic pain, which is characterized by hyperalgesia, allodynia, and spontaneous pain [1,2]. An increasing number of patients have been afflicted by neuropathic pain, which results in a cost of over 3 billion dollars in worldwide healthcare per year [3]. Currently, numerous drugs such as opioids, tricyclic antidepressants, anticonvulsant agents, and nonsteroidal anti-inflammatory drugs are applied for neuropathic pain therapy. However, these drugs still have various side effects or produce tolerance if they are used for an extended time, or they cannot meet the medical need [3,4,5]. Nowadays, acupuncture has been widely used for relieving various pathologic pains such as inflammatory pain [6,7] and neuropathic pain [8,9]. Electroacupuncture (EA), a modified acupuncture technique, has been shown to effectively relieve hyperalgesia [10,11] and allodynia [12,13,14]. However, the mechanism by which EA relieves neuropathic pain is still not fully understood.

Studies reported that neuropathic pain following nerve injury would result in excessive release of the central excitatory amino acids (EAAs, i.e., glutamate and aspartate) and an influx of Ca^2+^ in the spinal cord dorsal horn (SCDH) [15,16]. The release of Ca^2+^-dependent EAAs have been verified to be regulated by synaptotagmins, a family of membrane-trafficking proteins that are characterized by an N-terminal, transmembrane region, a variable linker, and two functional Ca^2+^ binding C2-domains [17]. Synaptotagmin 1 (Syt-1), a member of synaptotagmins, is widely distributed in central neuronal tissues. It is identified as a synaptic vesicle protein and a major Ca^2+^ sensor for regulating exocytosis or fine-tuning Ca^2+^-dependent neurotransmitter releases at presynaptic terminals [18,19]. Moreover, studies reported that Syt-1 is required for Ca^2+^ triggering of asynchronous neurotransmitter release and that Syt-1 knock-out mice exhibited a greatly reduced fast synchronous glutamate release in hippocampal neurons [19,20]. Previous studies have demonstrated that EA reduces glutamate levels at synaptic terminals through increasing glutamate/aspartate transporter and glutamate transporter-1 levels [21] and thereby relieve neuropathic pain in rats [9,22,23]. Hu et al. [24] used a high throughput sequencing technique for mRNAs in the periaqueductal gray of goats and found that EA induced differential expressions of Syt-1 mRNAs, demonstrating that Syt-1 could be involved in EA stimulation. However, whether Syt-1 participates in neuropathic pain and EA-induced analgesia has not been reported to date.

In the present study, spared nerve injury (SNI) rats were treated with antibody blockade, siRNA gene silencing, and lentivirus-mediated overexpression of spinal Syt-1 to confirm regulatory functions of Syt-1 for neuropathic pain. The distribution of Syt-1 in spinal neurons and glial cells was observed with the immunofluorescence method. The mechanical and thermal hypersensitivities were determined with the paw withdrawal threshold (PWT) and paw withdrawal latency (PWL), respectively. The mRNA and protein of Syt-1 were detected with qRT-PCR and Western blot, respectively. SNI rats were stimulated by EA to verify the involvement of Syt-1 in EA-induced analgesia. Our work contributes to further exploring the mechanism by which Syt-1 modifies nociception and EA-mediated alleviation of neuropathic pain and lay a foundation for developing a new strategy to intervene in this disorder.

## 2. Results

### 2.1. Syt-1 Is Up-Regulated Mainly in Spinal Lamina I-II after Neuropathic Pain

**Figure 1:**
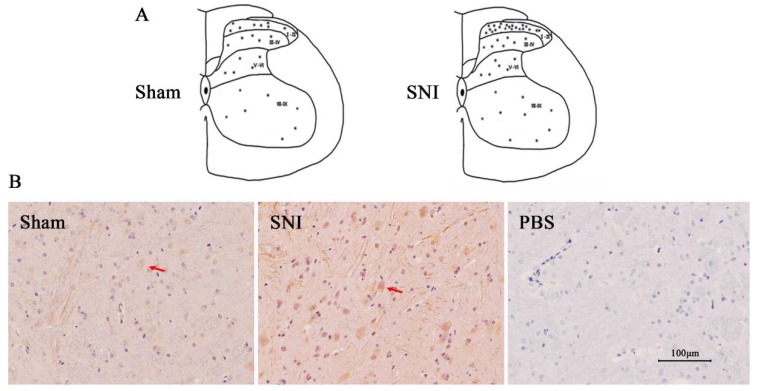
The distribution of Syt-1 immunoreactive cells in the spinal cord dorsal horn (SCDH). (**A**,**B**) The sketches and tissues (in laminae I-II) showed that SNI resulted in increased Syt-1 immunoreactive (Syt-1-IR), mainly in the laminae I-II of the SCDH. B shows the representative of immunohistochemistry from 3 animals. Red arrows show Syt-1 positive cells. Scale bars, 100 µm.

We first investigated the correlative relationship between Syt-1 and neuropathic pain. The result showed that Syt-1-IR cells were scattered in the spinal cord. Compared with Sham surgery, SNI caused increased Syt-1-IR, mainly in the laminae I–II of the SCDH (Figure 1).

### 2.2. Syt-1 Antibody Relieved SNI-Induced Neuropathic Pain

**Figure 2:**
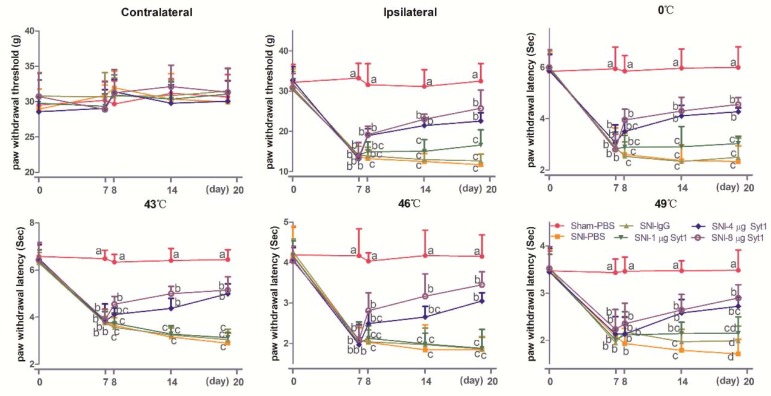
The effect of Syt-1 antibody on paw withdrawal thresholds (PWTs) and paw withdrawal latencies (PWLs) (Mean ± SD, *n* = 6). Rats in SNI+ different doses of Syt-1 antibody were intrathecally injected with 1, 4, or 8 µg antibody at day 8 and once every two days. SNI induced a decrease in PWLs (0, 43, 46, and 49 °C) and ipsilateral PWTs at day 7 to 20. Syt-1 antibody treatment caused increased PWLs (0, 43, 46, and 49 °C) and ipsilateral PWTs in a dose-dependent manner at day 8 to 20. Values with different letters (a, b and c) at the same day show different (*p* < 0.05); two-way ANOVA followed by Bonferroni test.

In order to study the effect of Syt-1 in the neuropathic pain, the effects of Syt-1 antibody on mechanical, hot, and cold sensitivities in SNI rats were investigated and showed in Figure 2 (Contralateral: Time × treatment, F_20, 120_ = 0.5632, *p* = 0.9305; time, F_3.422, 102.7_ = 2.033, *p* = 0.1056; treatment, F_5, 30_ = 0.3078, *p* = 0.9044. Ipsilateral: Time × treatment, F_20, 120_ = 8.752, *p* < 0.0001; time, F_3.180, 95.39_ = 118.2, *p* < 0.0001; treatment, F_5, 30_ = 109.0, *p* < 0.0001. 0 °C: Time × treatment, F_20, 120_ = 7.990, *p* < 0.0001; time, F_3.389, 101.7_ = 109.9, *p* < 0.0001; treatment, F_5, 30_ = 85.37, *p* < 0.0001. 43 °C: Time × treatment, F_20, 120_ = 11.60, *p* < 0.0001; time, F_3.803, 114.1_ = 169.6, *p* < 0.0001; treatment, F_5, 30_ = 135.1, *p* < 0.0001. 46 °C: Time × treatment, F_20, 120_ = 7.554, *p* < 0.0001; time, F_3.498, 104.9_ = 103.7, *p* < 0.0001; treatment, F_5, 30_ = 65.36, *p* < 0.0001. 49 °C: time × treatment, F_20, 120_ = 4.888, *p* < 0.0001; time, F_3.507, 105.2_ = 74.25, *p* < 0.0001; treatment, F_5, 30_ = 48.73, *p* < 0.0001). No difference was observed in contralateral PWTs among different doses of Syt-1 antibodies during the whole experiment. Compared with the Sham group, SNI caused a decrease (*p* < 0.05) in ipsilateral PWTs for mechanical stimulation and in PWLs for cold and hot stimulation at 0, 43, 46 and 49 °C at day 7 to 20. Syt-1 antibody treatment counteracts the SNI-induced decrease in PWLs and ipsilateral PWTs in a dose-dependent manner at day 8 to 20. Rats treated with 4 or 8 µg Syt-1 antibody showed greater (*p* < 0.05) ipsilateral PWTs and PWLs (0, 43, 46, and 49 °C) than those of SNI, SNI-IgG, or SNI-1 µg Syt-1 antibody group at day 14 to 20, but less (*p* < 0.05) than those of Sham group at day 8 to 20. There was no difference in ipsilateral PWTs and PWLs (0, 43, 46, and 49 °C) among SNI, SNI-IgG, and SNI-1 µg Syt-1 antibody groups during the experiment.

### 2.3. Syt-1 Knockdown Attenuated Neuropathic Pain

**Figure 3:**
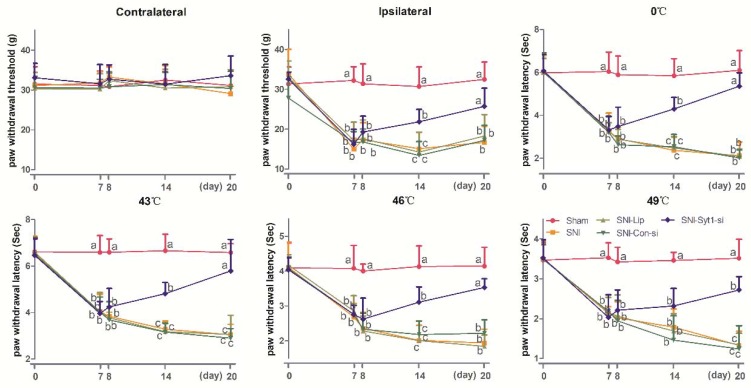
The effect of Syt-1 siRNA on PWTs and PWLs (Mean ± SD, *n* = 6). Rats in SNI-Lip, SNI-Con-si and SNI-Syt1-si groups were intrathecally injected with 15 µL lipofectamine, 10 µL lipofectamine + 5 µL control siRNA and 10 µL lipofectamine + 5 µL Syt-1 siRNA, respectively. Syt-1 siRNA induced increased ipsilateral PWTs and PWLs (0, 43, and 46 °C) at day 14 and 20 compared with SNI group. Values with different letters (a, b and c) at the same day show different (*p* < 0.05); two-way ANOVA followed by Bonferroni test.

The siRNA interference decreased Syt-1 expression and showed a similar effect on PWTs and PWTs as a Syt-1 antibody blockage. No difference was observed in contralateral PWTs among different treatments during the whole experiment. Compared with Sham surgery, SNI caused a decrease (*p* < 0.05) in ipsilateral PWTs, and PWLs (0, 43, 46, and 49 °C) at day 7 to 20. Syt-1 siRNA induced increased (*p* < 0.05) ipsilateral PWTs and PWLs (0, 43 and 46 °C) at day 14 and 20, and increased (*p* < 0.05) PWLs at 49 °C at day 20 compared with SNI group. There was no difference in ipsilateral PWTs and PWLs (0, 43, 46 and 49 °C) among SNI, SNI-Lip and SNI-Con-si groups (Figure 3. Contralateral: Time × treatment, F_16, 100_ = 0.4258, *p* = 0.9724; time, F_3.573, 89.32_ = 0.3636, *p* = 0.8131; treatment, F_4, 25_ = 0.7533, *p* = 0.5653. Ipsilateral: Time × treatment, F_16, 100_ = 6.803, *p* < 0.0001; time, F_3.418, 85.44_ = 76.32, *p* < 0.0001; treatment, F_4, 25_ = 98.61, *p* < 0.0001. 0 °C: Time × treatment, F_16, 100_ = 9.436, *p* < 0.0001; time, F_3.804, 95.09_ = 77.33, *p* < 0.0001; treatment, F_4, 25_ = 96.08, *p* < 0.0001. 43 °C: Time × treatment, F_16, 100_ = 8.501, *p* < 0.0001; time, F_3.591, 89.78_ = 84.37, *p* < 0.0001; treatment, F_4, 25_ = 85.80, *p* < 0.0001. 46 °C: Time × treatment, F_16, 100_ = 6.301, *p* < 0.0001; time, F_3.094, 77.36_ = 55.19, *p* < 0.0001; treatment, F_4, 25_ = 89.59, *p* < 0.0001. 49 °C: Time × treatment, F_16, 100_ = 1.251, *p* < 0.0001; time, F_1.087, 27.17_ = 7.774, *p* < 0.0001; treatment, F_4, 25_ = 1.360, *p* < 0.0001).

**Figure 4:**
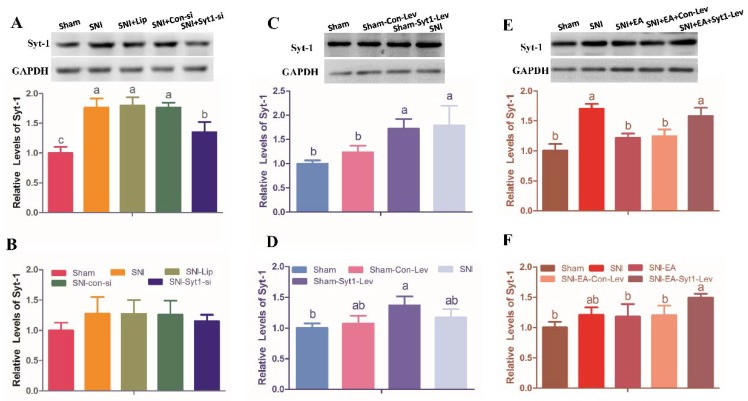
The effect of Syt-1 knockdown, Syt-1 overexpression or EA stimulations on Syt-1 expression (Mean ± SD, *n* = 6). (**A**,**B**) Syt-1 mRNA and protein levels were detected at day 20. Intrathecal injection of Syt-1 siRNA after SNI treatment decreased the protein of Syt-1, but unchanged its mRNA. (**C**,**D**) Intrathecal injection of Syt-1 activation particles shows the similar tendency of Syt-1 protein as SNI surgery. The mRNA and protein levels of Syt-1 in Sham-Syt1-Lev rats were higher than those in Sham group. (**E**,**F**) Rats in SNI-EA group showed decreased Syt-1 protein levels compared with rats in SNI group. Intrathecal injection of Syt-1 lentiviral activation particles induced an increase in Syt-1 protein and mRNA compared with EA treatment. No change was observed in Syt-1 mRNA among Sham, SNI, SNI-EA and SNI-Con-Lev groups. A, C, and D shows Syt-1 protein; B, E and F shows Syt-1 mRNA. Values with different letters (a, b and c) at the same day show different (*p* < 0.05); one-way ANOVA followed by Bonferroni test.

Compared with rats in Sham group, rats in SNI group showed enhanced (*p* < 0.05) Syt-1 protein. Syt-1 siRNA treatment decreased (*p* < 0.05) Syt-1 protein compared with SNI treatment. There was no difference in Syt-1 protein among SNI, SNI-Lip, and SNI-Con-si group, and in Syt-1 mRNA among different treatments. (Figure 4A,B).

### 2.4. Syt-1 Overexpression Causes Pain Hypersensitivity

**Figure 5:**
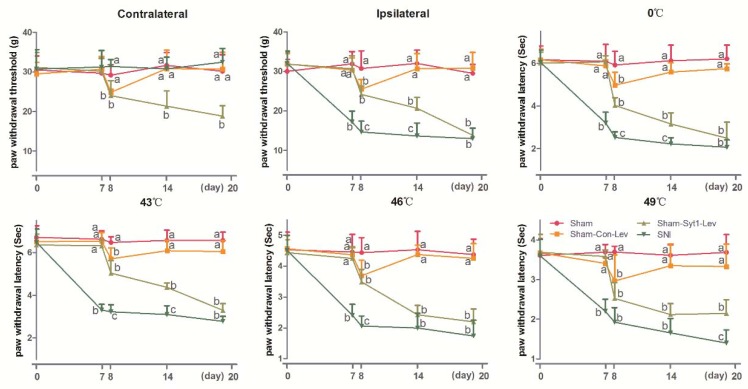
The effect of Syt-1 lentiviral activation particles on PWTs and PWLs (Mean ± SD, *n* = 6). Rats in Sham-Con-Lev and Sham-Syt1-Lev groups were intrathecally injected with 15 µL control lentiviral particles and Syt-1 lentiviral activation particles, respectively. Bilateral PWTs and PWLs (0, 43, 46 and 49 °C) in Sham-Syt1-Lev rats were less than those in Sham-Con-Lev at day 14 and 20. Ipsilateral PWTs and PWLs (0, 43, and 46 °C) of Sham-Syt1-Lev rats were decreased during the experiment, but not different from those of SNI rats at day 20. Values with different letters (a, b, and c) at the same day show different (*p* < 0.05); two-way ANOVA followed by Bonferroni test.

Figure 5 exhibited PWTs and PWLs at different temperatures in rats intrathecally administered with 15 µL of Syt-1 lentiviral activation particles or control lentiviral activation (Contralateral: Time × treatment, F_12, 80_ = 5.098, *p* < 0.0001; time, F_2.967, 59.34_ = 4.196, *p* = 0.0095; treatment, F_3, 20_ = 14.10, *p* < 0.0001. Ipsilateral: Time × treatment, F_12, 80_ = 14.81, *p* < 0.0001; time, F_3.534, 70.68_ = 37.21, *p* < 0.0001; treatment, F_3, 20_ = 119.4, *p* < 0.0001. 0 °C: Time × treatment, F_12, 80_ = 18.73, *p* < 0.0001; time, F_3.891, 77.82_ = 58.89, *p* < 0.0001; treatment, F_3, 20_ = 237.9, *p* < 0.0001. 43 °C: Time × treatment, F_12, 80_ = 22.18, *p* < 0.0001; time, F_2.921, 58.42_ = 66.34, *p* < 0.0001; treatment, F_3, 20_ = 247.6, *p* < 0.0001. 46 °C: Time × treatment, F_12, 80_ = 12.05, *p* < 0.0001; time, F_3.355, 67.11_ = 35.34, *p* < 0.0001; treatment, F_3, 20_ = 117.1, *p* < 0.0001. 49 °C: Time × treatment, F_12, 80_ = 8.038, *p* < 0.0001; time, F_2.953, 59.07_ = 27.22, *p* < 0.0001; treatment, F_3, 20_ = 126.2, *p* < 0.0001). There was no difference in bilateral PWTs and PWLs (0, 43, 46 and 49 °C) between the Sham group and Sham-Con-Lev group at day 14 and 20 of the experiment. SNI caused decreased (*p* < 0.05) ipsilateral PWTs and PWLs (0, 43, 46, and 49 °C), but did not change contralateral PWTs. Bilateral PWTs and PWLs in Sham-Syt1-Lev rats were less (*p* < 0.05) than those in Sham-Con-Lev at day 14 and 20. No difference was observed in ipsilateral PWTs and PWLs (0, 43, and 46 °C) between the Sham-Syt1-Lev and SNI group at day 20.

Syt-1 mRNA and protein levels were measured at day 20 (Figure 4C,D). The mRNA and protein levels of Syt-1 in Sham-Syt1-Lev rats were higher (*p* < 0.05) than those in Sham group. No difference was observed in Syt-1 mRNA and protein levels between the Sham-Syt1-Lev and SNI groups. There was no difference in Syt-1 mRNA levels among Sham, Sham-Con-Lev, and SNI group.

### 2.5. EA Attenuated Neuropathic Pain via Down-Regulating Syt-1

**Figure 6:**
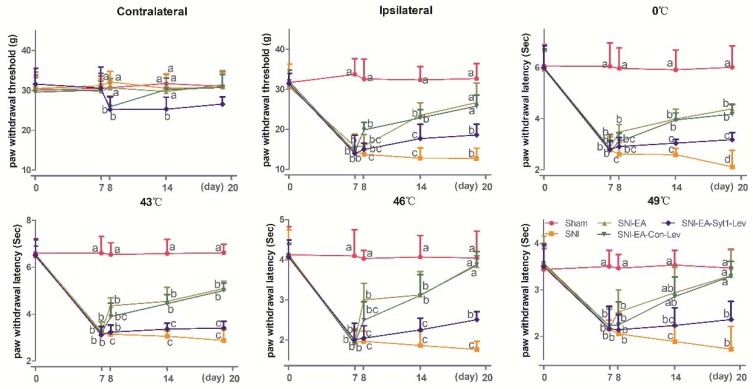
The effect of EA on PWTs and PWLs (Mean ± SD, *n* = 6). Rats in SNI-EA, SNI-EA-Con-Lev, or SNI-EA-Syt1-Lev groups were treated with EA at day 8, 10, 12, 14, 16, 18, and 20, for 7 times in total. Rats in the SNI-EA-Syt1-Lev or SNI-EA-Con-Lev groups were intrathecally injected with 15 µL of Syt-1 lentiviral activation particles or lentiviral control particles. The SNI-EA group showed increased ipsilateral PWTs, PWLs (0, 43, and 46 °C) at day 8 to 20 and PWL (49 °C) at day 20 compared with the SNI group. EA induced an increase in ipsilateral PWTs and PWLs at (0, 43, and 46 °C) at day 14 and 20 and in PWL (49 °C) at day 20, which was reversed by the intrathecal administration of Syt-1 lentiviral activation particles. Values with different letters (a, b, and c) on the same day show different (*p* < 0.05); two-way ANOVA followed by a Bonferroni test.

The effect of EA on neuropathic pain was shown in Figure 6 (Contralateral: Time × treatment, F_16, 100_ = 0.3082, *p* = 0.9950; time, F_3.786, 94.65_ = 0.4370, *p* = 0.7714; treatment, F_4, 25_ = 0.1861, *p* = 0.9435. Ipsilateral: time × treatment, F_16, 100_ = 8.937, *p* < 0.0001; time, F_3.252, 81.31_ = 69.01, *p* < 0.0001; treatment, F_4, 25_ = 97.72, *p* < 0.0001. 0 °C: time × treatment, F_16, 100_ = 8.336, *p* < 0.0001; time, F_3.021, 75.52_ = 94.44, *p* < 0.0001; treatment, F_4, 25_ = 127.8, *p* < 0.0001. 43 °C: Time × treatment, F_16, 100_ = 14.90, *p* < 0.0001; time, F_2.937, 73.42_ = 156.2, *p* < 0.0001; treatment, F_4, 25_ = 174.4, *p* < 0.0001. 46 °C: time × treatment, F_16, 100_ = 8.595, *p* < 0.0001; time, F_3.594, 89.84_ = 65.02, *p* < 0.0001; treatment, F_4, 25_ = 90.71, *p* < 0.0001. 49 °C: Time × treatment, F_16, 100_ = 4.790, *p* < 0.0001; time, F_3.672, 91.81_ = 30.69, *p* < 0.0001; treatment, F_4, 25_ = 33.75, *p* < 0.0001). No difference was observed in contralateral PWTs among Sham, SNI, and SNI + EA treatments during the whole experiment. Compared with Sham treatment, SNI induced a decrease (*p* < 0.05) in ipsilateral PWTs and PWLs (0, 43, 46, and 49 °C) at day 7 to 20. SNI-EA group showed an increase (*p* < 0.05) in ipsilateral PWTs and PWLs (0, 43, and 46 °C) at day 8 to 20, and in PWL (49 °C) at day 14 and 20 compared with SNI group. There is no difference in ipsilateral PWTs and PWLs between the SNI-EA and SNI-EA-Con-Lev groups. SNI-EA-Syt1-Lev rats showed a decrease (*p* < 0.05) in ipsilateral PWTs and PWLs (0, 43, and 46 °C) at day14 and 20, and in PWL (49 °C) at day 20 compared with those in SNI-EA and SNI-EA-Con-Lev rats. No difference was observed in ipsilateral PWTs and PWLs between the SNI-EA-Syt1-Lev and SNI rats.

**Figure 7:**
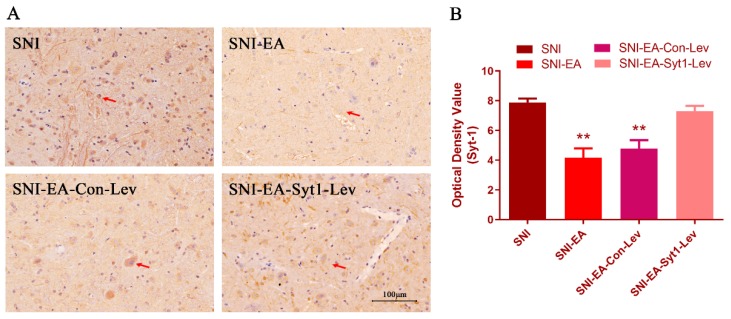
Effect of EA on Syt-1 immunoreactive cells in the laminae I-II of the SCDH (Mean ± SD, *n* = 3). (**A**) Decreased Syt-1-IR, which EA induced were apparently reversed by Syt-1 overexpression. Red arrows show Syt-1 positive cells. Scale bars, 100 µm. (**B**) The optical density values of the Syt-1-IR cells in the laminae I-II. The values in SNI and SNI-EA-Syt1-Lev were higher than those in SNI-EA and SNI-EA-Con-Lev. ** *p* < 0.01, vs. the SNI group; one-way ANOVA followed by Bonferroni test.

Syt-1 mRNA and protein levels were measured at day 20 (Figure 4E,F). SNI treatment enhanced (*p* < 0.05) Syt-1 protein expression compared with the Sham treatment. Rats in SNI-EA group showed decreased (*p* < 0.05) Syt-1 protein compared with rats in the SNI group. The Syt-1 protein in the SNI-EA-Syt1-Lev group was higher (*p* < 0.05) than that in SNI-EA group. Syt-1 lentiviral activation particle treatment induced an increase (*p* < 0.05) in Syt-1 mRNA compared with EA treatment. No change was observed in the Syt-1 mRNA among Sham, SNI, SNI-EA, or SNI-Con-Lev groups. IHC showed that decreased (*p* < 0.01) Syt-1-IR cells were induced by EA in laminae I-II of the SCDH of SNI rats, but reversed (*p* < 0.01) by the intrathecal injection of Syt-1 Lentiviral activation particles (Figure 7).

### 2.6. Syt-1 Was Expressed in Neurons and Glial Cells in the Spinal Cord Dorsal Horn

**Figure 8:**
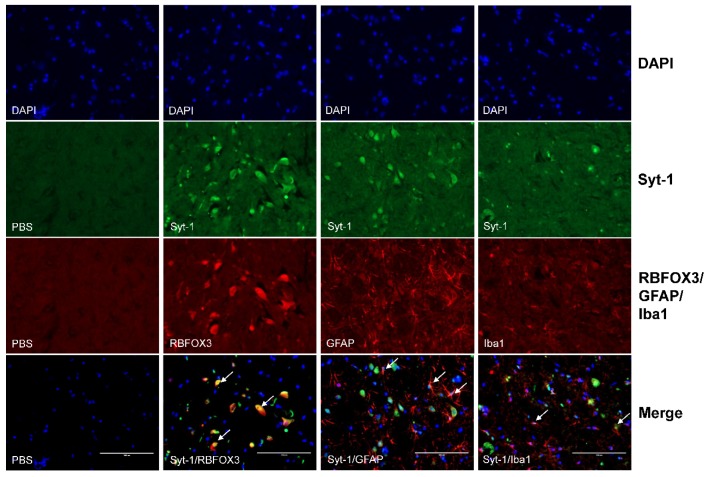
Colocalization of Syt-1 with the marker of neurons, astrocytes, or microglial cells in the SCDH. Co-labeling with RBFOX3 (a marker for neurons) and GFAP (a marker for astrocytes) antibodies showed that Syt-1-IR (in green) mainly exists in neurons and astrocytes (in red). Weak Syt-1-IR signal was observed in few microglial cells stained with Iba1 (a marker for microglial cells) antibodies. White arrows show the colocalization of Syt-1 with neurons, astrocytes, or microglial cells. Scale bars, 100 µm.

In double-labeling immunofluorescent trials, Syt-1-IR was observed to accumulate in neurons and most astrocytes, but in a few of microglial cells (Figure 8).

## 3. Discussion

Neuropathic pain, along with high incidence, complex pathogenesis, and lack of efficient treatments, attracts many researchers since it seriously affects patients’ life quality and increases social cost in recent years. Some models, such as chronic constriction injury, spared nerve ligation, and SNI, have been used for investigating the mechanisms underlying neuropathic pain. Compared with other models, SNI is simpler in manipulation and induces a more stable and apparent hypersensitivity [25,26,27]. In the present study, ipsilateral PWTs of SNI rats decreased from day 0 to day 7 and kept at a low level until day 20, and PWLs at different temperatures (0, 43, 46, and 49 °C) changed in a similar tendency as PWTs. These results were consistent with previous studies [9,25].

Syt-1 is widely distributed in the presynaptic vesicle membrane of the central neurons and affects the release of presynaptic neurotransmitters through participating in the synaptic vesicle fusion process in combination with soluble N-ethylmaleimide sensitive factor attachment protein receptor complex [28,29,30]. Gepper et al. [19] used homologous recombination to generate mice that carried a mutation in the Syt-1 gene and found that homozygotes died within 48 h after birth. Nishiki and Augustine [20] found that Syt-1 knock-out mice (heterozygous) exhibited a greatly reduced fast synchronous glutamate release in hippocampal neurons. Jia et al. [31] reported that prenatal stress led to a higher concentration of glutamate by enhancing Syt-1 expression in the hippocampus. Xiao and his colleagues [32] found that the Syt-1 level in the anterior temporal lobes of refractory epilepsy patients was significantly greater than that of non-refractory epilepsy and head trauma patients. Based on the endocytosis and exocytosis of Syt-1 in neurons, the authors inferred from their results that higher expression of Syt-1 might maintain and spread the seizure activity through facilitating the release of glutamate in the brain [32,33]. However, the roles of Syt-1 in other neuropathic conditions, including neuropathic pain, were rarely reported. In this study, the Syt-1 antibody or Syt-1 siRNA was intrathecally injected into SNI rats and found to cause an increase in ipsilateral PWTs and PWLs (0, 43, 46, and 49 °C). However, Syt-1 overexpression led to a decrease in ipsilateral PWTs and PWLs (0, 43, 46, and 49 °C) of rats. Our results indicate that the miRNA interference and antibody blockade of Syt-1 antagonize neural injury-induced allodynia and hyperalgesia; lentivirus-mediated overexpression of Syt-1 induces pain otherwise. Since Syt-1 was verified to regulate glutamate release [31], and the reduced glutamate has been confirmed to attenuate allodynia [34,35,36], our results suggest that Syt-1 may be involved in the development of neuropathic pain through facilitating glutamate release in SNI rats.

It is well known that EA can relieve diverse pains through the stimulation of acupoints [37,38,39,40]. In our study, EA increased PWTs in SNI rats, which is corresponding to the reports by Lau et al. [41] and Zeng et al. [9]. Numerous studies have demonstrated that EA-induced analgesia can be mediated by endogenous opioids [42,43,44]. However, microinjection of their antagonist, naloxone, only partly reverses the EA-induced analgesia under the pathological states [13,45], which suggests that EA antinociception is much more complex, and may involve other central substances or mechanisms besides opioid peptides. Glutamate plays a critical role in the development of mechanical allodynia and thermal hyperalgesia in the rats with nerve injury [15]. Studies have indicated that EA attenuates hyperalgesia and allodynia through reducing glutamate in the synaptic spaces in SNI rats [9,46]. In our study, SNI resulted in decreased ipsilateral PWTs and PWLs at different temperatures (0, 43, 46, and 49 °C) and increased spinal Syt-1 expression, which were reversed by EA. In addition, Syt-1 overexpression neutralized EA-induced Syt-1 reduction and reversed its analgesic effect in SNI rats. These results suggest that EA attenuates mechanical, hot, and cold nociception, probably through down-regulating spinal Syt-1. Since EA inhibits the release of glutamate in the spinal cord of SNI rats, it may attenuate neuropathic pain through acting on the spinal Syt-1-glutamate route. However, its related mechanism needs to be further studied in the future. In our experiment, EA decreased Syt-1 protein levels, but unchanged its mRNA level of SNI rats. This phenomenon may be attributed to the inconsistent time-course of the Syt-1 gene and protein expressions. In addition, Syt-1 expression may be regulated post-transcriptionally. MiRNAs, small endogenous non-coding RNA molecules, can be imprecisely complementary to their mRNA targets and inhibit protein synthesis while the targeted mRNA level remained unchanged. Cui et al. [47] sequenced miRNAs in the hypothalamus of EA-treated rats and found that Syt-1 mRNA transcripts were predicted as one of the putative target genes of 49 differentially expressed known miRNAs.

Qiu et al. [48] observed c-Fos, a reliable marker for activated nuclei, expressed in the spinal cord, and found that nociceptive stimulation mainly activated the neurons in laminae I and II of the SCDH. In the present study, Syt-1-IR cells were distributed in laminae I–VI of the SCDH. SNI surgery mainly increased Syt-1-IR in spinal laminae I–II, which was apparently reversed by EA. These results indicate that EA mediates inhibition of ascending nociception probably through downregulating Syt-1 in the spinal cord. Double-labelling immunofluorescent experiments demonstrated that Syt-1-IR mainly existed in spinal neurons and astrocytes. However, the roles of Syt-1 of spinal neurons or astrocytes in EA-induced analgesia need to be investigated.

## 4. Materials and Methods

### 4.1. Animals

Adult female Sprague-Dawley rats of 240 ± 20 g body weight (BW) were provided by Hubei Provincial Center for Disease Control and Prevention (No. 42000600017748). They were housed 6 per cage with food pellets and water ad libitum. The environment was maintained with a 12 h dark/light cycle (7:00 a.m. to 19:00 p.m.) and kept at 24 ± 2 °C. All rats were acclimatized to the environment for 1 week before the experiment. All rats included in this study were initially in a diestrus phase, which was confirmed by vaginal cytology [49]. This study adhered to the guidelines of the Committee for Research and Ethical Issues of the International Association for the Study of Pain. The protocol was approved by the Animal Care and Use Committee of Huazhong Agricultural University (HZAURA-2018-015) on 26 November 2018.

### 4.2. Groups and Design

To determine the effect of neuropathic pain on spinal Syt-1 distribution, 6 rats subjected to a sham or SNI surgery procedure (*n* = 3). The rats were euthanized at day 7. Their lumbar segments (L4–L6) of the spinal cords were taken out and stored in 10% formalin for immunohistochemistry (IHC).

**Figure 9:**
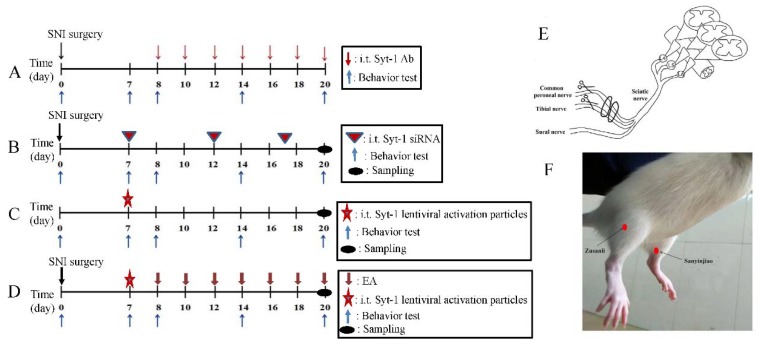
The schematic diagram for the experimental design. (**A**) The diagram for Syt-1 antibody intrathecal injection. (**B**) The diagram for Syt-1 siRNA intrathecal injection. (**C**) The diagram for Syt-1 lentiviral activation particles intrathecal injection. (**D**) The diagram for electroacupuncture (EA) stimulation after spared nerve injury (SNI) surgery. (**E**) The methods for the SNI model: A ligation of the common peroneal and tibial nerves was conducted with a 5.0 silk, leaving the sural nerve intact. Then, the two ligated nerves were sectioned distal to the ligation with 2–4 mm of the distal nerve stump left. (**F**) EA acupoints: Bilateral acupoints of “Zusanli” and “Sanyinjiao”. i.t. means intrathecal injection.

To detect the effect of Syt-1 antibody on nociception, 36 rats were randomly divided into 6 groups of 6 rats each: Sham, SNI, SNI + IgG, SNI + 1 µg Syt-1 antibody(SNI-1 µg Syt-1), SNI + 4 µg Syt-1 antibody (SNI-4 µg Syt-1), and SNI + 8 µg Syt-1 antibody (SNI-8 µg Syt-1). SNI-treated rats suffered SNI surgery. Rats in Sham rats suffered sham surgery (without ligation of any branches of the sciatic nerve). Sham and SNI groups were intrathecally administered with phosphate buffer saline (PBS). Rats in the the SNI + IgG group were intrathecally administered with Isotype IgG (Boster, WH, China). Three groups of SNI rats were intrathecally injected with 1, 4, or 8 μg Syt-1 antibody (R&D Systems, Minneapolis, MN, USA). The volume of intrathecally injected solutions was 20 µL. These reagents (PBS, IgG, and Syt-1 antibody) were given at day 8, 10, 12, 14, 16, 18, and 20 after the SNI surgery, for a total of 7 times. Mechanical, hot, and cold hypersensitivities were detected at day 0 and 7, and at 30 min after the intrathecal injection at day 8, 14, and 20 (Figure 9A).

**Figure 10:**
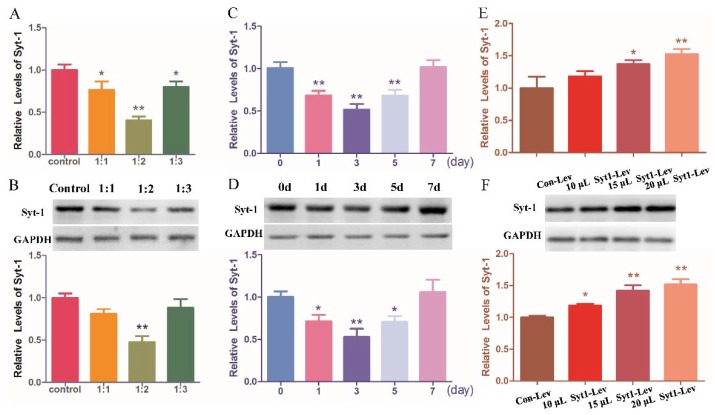
Exploration of the optimal conditions for Syt-1 interference and overexpression (Mean ± SD, *n* = 3). (**A**,**B**) Five microliters of Syt-1 siRNA in mixture with 10 µL lipofectamine (1:2) caused less Syt-1 mRNA and protein expression compared with the other ratios; * *p* < 0.05, ** *p* < 0.01, vs. control group. (**C**,**D**) After a bolus administration of the optimal Syt-1 siRNA vs. lipofectamine ratio (1:2), decreased Syt-1 expression lasted for 5 days at least; * *p* < 0.05, ** *p* < 0.01, vs. 0 d group. (**E**,**F**) Intrathecal injection of 15 µL Syt-1 lentiviral activation particles caused increased Syt-1 mRNA and protein compared with control lentiviral particles; * *p* < 0.05, ** *p* < 0.01, vs. Con-Lev group. A, C, and E show Syt-1 mRNA; B, D, and F show Syt-1 protein. In the control (A and B), phosphate buffer saline (PBS) instead of the Syt-1 siRNA mixture was intrathecally administered. The significance of differences was calculated with a one-way ANOVA followed by a Bonferroni test.

The effect of Syt-1 knockdown on neuropathic pain was investigated with Syt-1 siRNA. The ratio of Syt-1 siRNA to lipofectamine^TM^ 3000 transfection reagent (Invitrogen, Carlsbad, CA, USA) (V:V) and the action time of Syt-1 siRNA was determined in the pretest. Firstly, 12 rats were intrathecally injected with the mixture (5 µL Syt-1 siRNA with 5, 10, or 15 µL lipofectamine) or 15 µL PBS (*n* = 3), and euthanized at 72 h after injection to determine the ratio of Syt-1 siRNA to lipofectamine (V:V). Five microliters of Syt-1 siRNA (Santa Cruz, Dallas, TX, USA) in the mixture with 10 µL lipofectamine was found to be the most optimal ratio for the interference with Syt-1 expression (Figure 10A,B). Then, 15 rats were intrathecally administered with 15 µL of the mixture (5µL Syt-1 siRNA + 10 µL lipofectamine) and euthanized at 0, 1, 3, 5, or 7 d for determining Syt-1 siRNA action time (*n* = 3). Syt-1 siRNA action was found to last for 5 days (Figure 10C,D). To determine the effect of Syt-1 silencing on neuropathic pain in the formal test, 30 rats were randomly classified into 5 groups of 6 rats each: Sham, SNI, SNI + lipofectamine (SNI-Lip), SNI + lipofectamine mixture with control siRNA (SNI-Con-si), and SNI + lipofectamine mixture with Syt-1 siRNA (SNI-Syt1-si). Rats in the SNI-Lip, SNI-Con-si, and SNI-Syt1-si groups were intrathecally injected with 15 µL lipofectamine, 10 µL lipofectamine + 5 µL control siRNA, and 10 µL lipofectamine + 5 µL Syt-1 siRNA, respectively, at day 7, 12, and 17 after the surgery. Mechanical, hot, and cold hypersensitivities were detected at day 0, 7, 8, 14, and 20 (Figure 9B).

Syt-1 overexpression was conducted through clustered regularly interspaced short palindromic repeats (CRISPR) Lenti Activation Systems (Santa Cruz, Dallas, TX, USA), which can activate endogenous gene transcription using a robust synergistic activation mediator (SAM) system. In the SAM system, the catalytic domains of Cas9 have been deactivated, and the resulting dCas9 was fused to a transcription activation domain (VP64). Directed by a target-specific guide RNA (sgRNA), the dCas9-VP64-sgRNA complex targets the 284 bp region from the Transcriptional Start Site (TSS) of endogenous genes to upregulate gene expression of Syt-1.

To determine the optimal dosage of lentiviral activation particles for Syt-1 overexpression, 12 rats were intrathecally injected with Syt-1 lentiviral activation particles (10, 15, or 20 µL) or 15 µL control lentiviral particles (15 µL) (*n* = 3) and euthanized at 7 d after injection. Fifteen microliters of Syt-1 lentiviral activation particles were considered to be the optimal dosage because Syt-1 mRNA and protein levels increased in a dose-dependent manner after it was intrathecally injected (Figure 10E,F). To determine the effect of Syt-1 overexpression on neuropathic pain, 24 rats were randomly allocated into the following 4 groups of 6 rats per group: Sham, Sham + control lentiviral particles (Sham-Con-Lev), Sham + Syt-1 lentiviral activation particles (Sham-Syt1-Lev), and SNI. Mechanical, hot, and cold hypersensitivities were detected at day 0, 7, 8, 14, and 20. Rats in Sham-Con-Lev and Sham-Syt1-Lev group were intrathecally injected with 15 µL of control lentiviral particles and Syt-1 lentiviral activation particles, respectively, immediately after the behavior tests at day 7 (Figure 9C).

To further explore the role of Syt-1 in EA, 30 rats were randomly divided into 5 groups of 6 rats each: Sham, SNI, SNI + EA (SNI-EA), SNI + EA+ control lentiviral particles (SNI-EA-Con-Lev), and SNI + EA + Syt-1 lentiviral activation particles (SNI-EA-Syt1-Lev). Rats in SNI-EA, SNI-EA-Con-Lev, or SNI-EA-Syt1-Lev groups were treated with EA at day 8, 10, 12, 14, 16, 18, and 20, for 7 times in total, after SNI surgery. Rats in the Sham or SNI group were restrained in the same manner as the EA-treated rats. Mechanical, hot, and cold hypersensitivities were detected at day 0, 7, 8, 14, and 20 of the experiment. Rats in SNI-EA-Con-Lev and SNI-EA-Syt1-Lev groups were intrathecally injected with 15 µL of control lentiviral particles and Syt-1 lentiviral activation particles, respectively, immediately after behavior tests at day 7 of the experiment (Figure 9D). After PWL measurements, 6 rats from each group were sacrificed, and their spinal cord L4–L6 were taken out and divided into 2 parts; the anterior part was stored in 10% formalin for IHC, and the posterior part was weighed and frozen in liquid nitrogen for Western blot and qRT-PCR analysis.

Syt-1 has been reported to express in neurons. To determine whether it was also expressed in glial cells (astrocytes and microglial cells), 3 rats were euthanized. The spinal cord L4–L6 was taken out for double-labeling immunohistofluorescent experiments with markers of neurons (RBFOX3), astrocytes (GFAP), and microglia (Iba1).

### 4.3. SNI Rat Preparation

The SNI procedure was conducted according to a previous report [25]. Briefly, under anesthesia with pentobarbital sodium (30 mg/kg), an incision was made on the skin on the right lateral thigh, and the biceps femoris muscle was directly dissected to expose 3 terminal branches of the sciatic nerve: The sural, common peroneal, and tibial nerves. The tibial and common peroneal nerves were ligated with a 5-0 silk, leaving the sural nerve intact. Then the 2 ligated nerves were sectioned distal to the ligation with 2–4 mm of the distal nerve stump left (Figure 9E). Great care was given to rats to avoid contacting or stretching the intact sural nerve. The sciatic nerve of the Sham group was exposed with the 3 branches intact. The muscles and skin of the rats were then closed, respectively. The rats with self-mutilation or paralysis after the surgery were abnegated in the experiment.

### 4.4. Intrathecal Cannulation

Following SNI surgery, rats were prepared for intrathecal placement of a polyethylene catheter (PE-10) according to previous reports [9,50]. Briefly, a stretched PE-10 intrathecal catheter (16–18 cm, Smith Medical, Kent, UK) was inserted into the lumbar enlargement at the section of the L4–L6 in the spinal cord through a 20-gauge guide cannula (0.9 × 35 mm) about 3 cm beyond its tip. The other end of the catheter was tunneled through the skin to exit in the occipital region. The rats were injected with 20 µL of 2% lidocaine (Shandonghualu Pharmaceutical CO., LTD., JN, China) through the end of the catheter to confirm the success of its intrathecal placement. Animals lost sensation of double lower limbs and showed lameness within 30 s, followed by motor functions recovered in approximately 30 min, showing that the catheter was located stably in the subarachnoid space. Rats with overt signs of spinal cord damage such as paralysis or lameness 1 week after the surgery were excluded in the experiment. A total of 4 rats with spinal cord damage had been replaced by the spare rats to guarantee 6 rats/group. Different doses of reagents (anti-Syt-1 antibody, Syt-1 siRNA, Syt-1 overexpression reagents, etc.) were delivered and followed by 15 µL of saline solution into the catheter with a micro-syringe needle (Gaoge, SH, China) at a rate of 10 µL/min.

### 4.5. Mechanical Hypersensitivity

The hypersensitivity in response to mechanical stimuli was assessed through quantifying the PWT of ipsilateral and contralateral hind paws [51]. Animals were firstly adapted to the test room (12 × 22 × 18 cm^3^) through quietly standing on a metal mesh for 20 min. PWTs of the hind paws were measured with an electronic von Frey aesthesiometer (ZS-CITY Beijing Zhongshi-Dichuang Science and Technology Development Co, Ltd. BJ, China) with a force transducer fitted with a 0.5 mm diameter polypropylene rigid tip. A von Frey of normally innocuous stimulation was applied vertically to the mid-plantar surface of the paws with a series of ascending pressures. The lowest force that caused the withdrawal response was automatically recorded by the aesthesiometer. PWT tests were repeated 3 times with a 5 min interval. The mean values were calculated from 3 repeated PWTs.

### 4.6. Cold and Hot Hypersensitivities

Thermal hypersensitivity was evaluated through quantifying paw withdrawal latency (PWL) in hind paws. The animals were placed on the cold and hot-plate surface of a hot plate tester (ZS-CITY Beijing Zhongshi-Dichuang Science and Technology Development Co, Ltd. BJ, China) set at defined temperatures (0, 43, 46, and 49 °C) [52]. The latency of response (in seconds) was determined until the hind paw was licked or lifted up. The cutoff was adjusted for the temperature to avoid tissue damage (25 s for 0 °C, 30 s for 43 °C, 20 s for 46 °C, and 15 s for 49 °C). Three trials were performed at 5 min intervals. The averages were calculated from 3 repeated values.

### 4.7. Electroacupuncture Application

Rats were given EA stimulation at a fixed time of day (9:00 a.m.), as previously described [53]. Each rat was fixed loosely in a polyethylene holder with its hind legs and tail exposed. The needles and acupoint sites were disinfected with 75% ethanol. Bilateral acupoints of “Zusanli” (ST36, a notch 4 mm lateral to the anterior tuber point of the tibia, 6–7 mm depth) and “Sanyinjiao” (SP6, 3 mm proximal to the middle malleolus at the posterior border of the tibia, 4–5 mm depth) were treated with stainless-steel needles (0.30 mm in diameter, 13 mm in length) (Figure 9E). Each pair of needles were connected to WQ-6F Electronic Acupuncto-scope (Beijing Xindonghua Electronic Instrument Co., Ltd., BJ, China) with wires. According to Zeng et al.’s method [9], EA stimuli parameters were set as follows: Square waves, 2 Hz in frequency, and 1–2–3 mA (initial strength 1 mA, increased by 1 mA every 10 min) for a total of 30 min.

### 4.8. Sample Collection

Rats were euthanized on the last day of the experiment with an overdose of sodium pentobarbital after mechanical, hot, and cold hypersensitivities were measured. Their spinal cord L4–L6 were taken out, weighed, and frozen in liquid nitrogen for Western blot and qRT-PCR analysis.

Three rats for the observation of Syt-1 distribution were deeply anesthetized with sodium pentobarbital (40 mg/kg), and intracardially perfused with cold 4% paraformaldehyde. The spinal cord L4–6 was removed for immunofluorescence.

### 4.9. Immunohistochemistry

The primary antibodies used for this study were the mouse anti-Syt-1 antibody (8 μg/mL, R&D, MN, Minneapolis, USA), rabbit anti-RBFOX3, rabbit anti-GFAP, and rabbit anti-Iba1 antibodies (1:200, Abclonal, WH, China). Secondary antibodies used were HRP-conjugated goat anti-mouse (Boster, WH, China), Cy3 labeled goat anti-rabbit (1:500, Boster, WH, China), and FITC-conjugated goat anti-mouse antibodies (1:500, Bioss, BJ, China).

For IHC, the spinal cord L4–L6 was taken out and preserved in 4% paraformaldehyde diluted with 0.1 M PBS at room temperature and embedded in paraffin with their surface facing up. Each of the blocks was consecutively sectioned with a thickness of 5 µm. Antigens were retrieved with 0.01 M citric acid (pH 6.0) for 15 min at 90–97 °C in a microwave oven. After being treated in 3% H_2_O_2_ for blocking the endogenous peroxidase for 30 min, the sections were incubated with 5% BSA for 30 min to reduce non-specific binding. The slides were incubated with the primary antibody at 4 °C for 12 h and incubated with the secondary antibody at room temperature for 30 min. Immunoreaction was visualized using 3, 3’-diaminobenzidine.

For fluorescent IHC, the spinal cords were cryoprotected in 30% sucrose in PBS and embedded in TissuTek. Cryostat sections (10 µm) were collected on slides. The sections were incubated in 5% BSA at room temperature for 30 min and incubated with the primary antibody at 4 °C for 12 h. After being washed with PBS, sections were incubated with the secondary antibody at room temperature for 2 h and stained with DAPI (1:2000, Beyotime, SH, China).

The sections stained with IHC were observed under a light microscope (Nikon ECLIPSE80I, Nikon Corporation, TKY, Japan) connected to a video-based and computer-linked system (high-resolution pathological image analysis system 1000, Wuhan QianpingLtd., WH, China). The optical density values of the Syt-1 immunoreactive (Syt-1-IR) cells in the laminae I-II of the SCDH were calculated with the Image-Pro plus 6.0 system (Media Cybernetics, Inc., Bethesda, MD, USA). The sections stained with immunofluorescence were analyzed using an Invitrogen EVOS FL Auto Cell Imaging System (Thermo Fisher Scientific, Waltham, MA, USA).

### 4.10. Western Blotting

The frozen spinal cord L4–6 was ground and mixed with cold RIPA Buffer (200 µL per 20 mg of tissue) (Beyotime Biotech, SH, China). Forty micrograms of proteins were subjected to 10% SDS-PAGE and transferred to a PVDF membrane using a minigel and mini transblot apparatus (Bio-Rad, Hercules, CA, USA). After being incubated in 5% nonfat milk containing 0.1% Tween-20 for 2 h, the membrane was infiltrated with the primary mouse anti-Syt-1 antibody (4 µg/mL, R&D Systems, Minneapolis, MN, USA) at 4 °C overnight. The membrane was washed in Tris-Buffered saline Tween-20 (TBST) 3 times and incubated in the goat anti-mouse secondary antibody (1:3000, Servicebio, WH, China) at 37 °C for 1 h. GAPDH (1:1000, Servicebio, WH, China) was used as the protein control. The antigen-antibody complex was reacted with a horseradish peroxidase substrate (Millipore, Burlington, MA, USA) and visualized using the ImageQuant LAS 4000 min CCD camera (GE Healthcare, Boston, MA, USA). The bands were analyzed by Quantity One software (Bio-Rad, Hercules, CA, USA). Values of Syt-1 were represented as the optical density ratio of the target protein bands to the related GAPDH bands.

### 4.11. QRT-PCR

Total RNA of the spinal cord L4–6 was extracted using Trizol reagent (Invitrogen, Carlsbad, CA, USA) and subsequently used for cDNA synthesis with a First Strand cDNA Synthesis Kit (TOYOBO, OSA, Japan). SYBR Premix Ex Taq II (Takara Bio Inc, SIGA, Japan) was used for amplifying the Syt-1 gene with Step One Real-Time PCR System (Applied Biosystems, Carlsbad, CA, USA). The primers specific for the Syt-1 gene (NCBI number: 25716) were as follows: 5’-AACACACTCAACCCCTACTAC AA-3’ (forward); 5’-GGCATCAACCTCCTCCTCTA-3’ (reverse). Rat GAPDH primers were purchased from Sangon Biotech Co., Ltd., Shanghai, China. The mRNAs of Syt-1 relative to GAPDH were quantified with 2^-ΔCt^ method, where ΔCt = Ct _target gene_−Ct _GAPDH._

### 4.12. Statistics

All data were presented as the Mean ± SD. Statistical analysis was performed using SPSS 17.0 statistical software (IBM Co., Armonk, NY, USA). One-way analysis of variance was used to analyze the expression levels of proteins and mRNAs (Figure 4, Figure 7 and Figure 10). The PWTs and PWLs (Figure 2, Figure 3, Figure 5 and Figure 6) were analyzed with a two-way analysis of repeated variance (ANOVA). A Bonferroni test was used when significant differences were found. *p* < 0.05 was considered significant.

## 5. Conclusions

Our research, for the first time, demonstrated that Syt-1 exacerbates neuropathic pain and that EA alleviates neuropathic pain through down-regulating spinal Syt-1. Since Syt-1 could bind Ca^2+^ and trigger synaptic vesicle exocytosis of neurotransmitters, EA exerts its antinociception probably through down-regulating Syt-1 and thereby inhibiting the release of excitatory neurotransmitters such as glutamate. Because supraspinal nuclei have complex functions and their neuronal axons communicate between different brain structures, the roles of supraspinal Syt-1 in EA-induced relief of neuropathic pain were not involved in this study. EA was found to alleviate neuropathic pain through down-regulating spinal Syt-1 in our study, but the way EA regulates the expression of Syt-1 remains unclear. The limited data in the study only established a preliminary link between the Syt-1-induced pronociception and EA-mediated antinociception under the neuropathic states. Anyway, the current findings offer a new avenue for understanding the mechanisms by which EA relieves neuropathic pain.

## Abbreviations

EA	Electroacupuncture
Syt-1	Synaptotagmin 1
SNI	Spared nerve injury
PWT	Paw withdrawal threshold
PWL	Paw withdrawal latency
EAAs	Excitatory amino acids
ST36	Zusanli
SP6	Sanyinjiao
SCDH	Spinal cord dorsal horn
IHC	Immunohistochemistry
IR	Immunoreactive
PBS	Phosphate buffer saline

## References

[1] Kajander K.C., Bennett G.J. (1992). Onset of a painful peripheral neuropathy in rat: A partial and differential deafferentation and spontaneous discharge in A beta and A delta primary afferent neurons. J. Neurophysiol..

[2] Woolf C.J., Mannion R.J. (1999). Neuropathic pain: Aetiology, symptoms, mechanisms, and management. Lancet.

[3] Butera J.A. (2007). Current and emerging targets to treat neuropathic pain. J. Med. Chem..

[4] Tawfik V.L., Nutile-McMenemy N., LaCroix-Fralish M.L., Deleo J.A. (2007). Efficacy of propentofylline, a glial modulating agent, on existing mechanical allodynia following peripheral nerve injury. Brain Behav. Immun..

[5] Muthuraman A., Singh N., Jaggi A.S., Ramesh M. (2014). Drug Therapy of Neuropathic Pain: Current Developments and Future Perspectives. Curr. Drug Targets.

[6] Hu M.-L., Zhou F.-Y., Liu J.-J., Ding Y., Zhong J.-M., Ding M.-X. (2017). Electroacupuncture Inhibits the Activation of p38MAPK in the Central Descending Facilitatory Pathway in Rats with Inflammatory Pain. Evid.-Based Complementary Altern. Med..

[7] Liao H.Y., Hsieh C.L., Huang C.P., Lin Y.W. (2017). Electroacupuncture Attenuates Induction of Inflammatory Pain by Regulating Opioid and Adenosine Pathways in Mice. Sci. Rep..

[8] Omana I., Olvera V., Santos P., Calderon J.L. (1994). Naloxone prevents reduction of pain responses evoked by acupuncture in neuropathic rats. Proc. West. Pharmacol. Soc..

[9] Zeng J., Cui L.Y., Feng Y., Ding M.X. (2016). Electroacupuncture relieves neuropathic pain via upregulation of glutamate transporters in the spinal cord of rats. Neurosci. Lett.

[10] Dai Y., Kondo E., Fukuoka T., Tokunaga A., Miki K., Noguchi K. (2001). The effect of electroacupuncture on pain behaviors and noxious stimulus-evoked Fos expression in a rat model of neuropathic pain. J. Pain Off. J. Am. Pain Soc..

[11] Dong Z.Q., Ma F., Xie H., Wang Y.Q., Wu G.C. (2006). Down-regulation of GFRalpha-1 expression by antisense oligodeoxynucleotide attenuates electroacupuncture analgesia on heat hyperalgesia in a rat model of neuropathic pain. Brain Res. Bull..

[12] Huang C., Li H.T., Shi Y.S., Han J.S., Wan Y. (2004). Ketamine potentiates the effect of electroacupuncture on mechanical allodynia in a rat model of neuropathic pain. Neurosci. Lett..

[13] Hwang B.G., Min B.I., Kim J.H., Na H.S., Park D.S. (2002). Effects of electroacupuncture on the mechanical allodynia in the rat model of neuropathic pain. Neurosci. Lett..

[14] Wan J., Ding Y., Tahir A.H., Shah M.K., Janyaro H., Li X., Zhong J., Vodyanoy V., Ding M. (2017). Electroacupuncture Attenuates Visceral Hypersensitivity by Inhibiting JAK2/STAT3 Signaling Pathway in the Descending Pain Modulation System. Front. Neurosci..

[15] Kawamata M., Omote K. (1996). Involvement of increased excitatory amino acids and intracellular Ca2+ concentration in the spinal dorsal horn in an animal model of neuropathic pain. Pain.

[16] Davar G., Hama A., Deykin A., Vos B., Maciewicz R. (1991). MK-801 blocks the development of thermal hyperalgesia in a rat model of experimental painful neuropathy. Brain Res..

[17] Sudhof T.C. (2002). Synaptotagmins: Why so many?. J. Biol. Chem..

[18] Fernández-Chacón R., Königstorfer A., Gerber S.H., García J., Matos M.F., Stevens C.F., Brose N., Rizo J., Rosenmund C., Südhof T.C. (2001). Synaptotagmin I functions as a calcium regulator of release probability. Nature.

[19] Geppert M., Goda Y., Hammer R.E., Li C., Rosahl T.W., Stevens C.F., Südhof T.C. (1994). Synaptotagmin I: A major Ca2+ sensor for transmitter release at a central synapse. Cell.

[20] Nishiki T., Augustine G.J. (2004). Synaptotagmin I synchronizes transmitter release in mouse hippocampal neurons. J. Neurosci..

[21] Cui L., Ding Y., Zeng J., Feng Y., Li M., Ding M. (2016). Spinal Glutamate Transporters Are Involved in the Development of Electroacupuncture Tolerance. Int. J. Mol. Sci..

[22] al-Ghoul W.M., Li Volsi G., Weinberg R.J., Rustioni A. (1993). Glutamate immunocytochemistry in the dorsal horn after injury or stimulation of the sciatic nerve of rats. Brain Res. Bull..

[23] Sung B.K., Lim G., Mao J.R. (2003). Altered expression and uptake activity of spinal glutamate transporters after nerve injury contribute to the pathogenesis of neuropathic pain in rats. J. Neurosci..

[24] Hu M.L., Zhu H.M., Zhang Q.L., Liu J.J., Ding Y., Zhong J.M., Vodyanoy V., Ding M.X. (2018). Exploring the Mechanisms of Electroacupuncture-Induced Analgesia through RNA Sequencing of the Periaqueductal Gray. Int. J. Mol. Sci..

[25] Decosterd I., Woolf C.J. (2000). Spared nerve injury: An animal model of persistent peripheral neuropathic pain. Pain.

[26] Bennett G.J., Xie Y.K. (1988). A peripheral mononeuropathy in rat that produces disorders of pain sensation like those seen in man. Pain.

[27] Kim S.H., Chung J.M. (1992). An experimental model for peripheral neuropathy produced by segmental spinal nerve ligation in the rat. Pain.

[28] Tucker W.C., Chapman E.R. (2002). Role of synaptotagmin in Ca(2+)-triggered exocytosis. Biochem. J..

[29] Bhalla A., Chicka M.C., Tucker W.C., Chapman E.R. (2006). Ca2+-synaptotagmin directly regulates t-SNARE function during reconstituted membrane fusion. Nat. Struct. Mol. Biol..

[30] Dai H., Shen N., Arac D., Rizo J. (2007). A quaternary SNARE-synaptotagmin-Ca2+-phospholipid complex in neurotransmitter release. J. Mol. Biol..

[31] Jia N., Yang K., Sun Q., Cai Q., Li H., Cheng D., Fan X., Zhu Z. (2010). Prenatal stress causes dendritic atrophy of pyramidal neurons in hippocampal CA3 region by glutamate in offspring rats. Dev. Neurobiol..

[32] Xiao Z., Gong Y., Wang X.F., Xiao F., Xi Z.Q., Lu Y., Sun H.B. (2009). Altered expression of synaptotagmin I in temporal lobe tissue of patients with refractory epilepsy. J. Mol. Neurosci. Mn.

[33] Proper E.A., Hoogland G., Kappen S.M., Jansen G.H., Rensen M.G.A., Schrama L.H., van Veelen C.W.M., van Rijen P.C., van Nieuwenhuizen O., Gispen W.H. (2002). Distribution of glutamate transporters in the hippocampus of patients with pharmaco-resistant temporal lobe epilepsy. Brain.

[34] Brann D.W. (1995). Glutamate: A major excitatory transmitter in neuroendocrine regulation. Neuroendocrinology.

[35] Liaw W.J., Stephens R.L., Binns B.C., Chu Y.C., Sepkuty J.P., Johns R.A., Rothstein J.D., Tao Y.X. (2005). Spinal glutamate uptake is critical for maintaining normal sensory transmission in rat spinal cord. Pain.

[36] Yan L.-p., Wu X.-t., Yin Z.-y., Ma C. (2011). Effect of electroacupuncture on the levels of amino acid neurotransmitters in the spinal cord in rats with chronic constrictive injury. Zhen Ci Yan Jiu = Acupunct. Res..

[37] Cui L.-Y., Guo N.-N., Li Y.-L., Li M., Ding M.-X. (2017). Analgesic and physiological effect of electroacupuncture combined with epidural lidocaine in goats. Vet. Anaesth. Analg..

[38] Hu M.L., Qiu Z.Y., Hu K., Ding M.X. (2016). Analgesic Neural Circuits Are Activated by Electroacupuncture at Two Sets of Acupoints. Evid.-Based Complementary Altern. Med. Ecam.

[39] Shah Z., Hu M.L., Qiu Z.Y., Zhou F.Y., Zeng J., Wan J., Wang S.W., Zhang W., Ding M.X. (2016). Physiologic and biochemical effects of electroacupuncture combined with intramuscular administration of dexmedetomidine to provide analgesia in goats. Am. J. Vet. Res..

[40] Liu D.-M., Zhou Z.-Y., Ding Y., Chen J.-G., Hu C.-M., Chen X., Ding M.-X. (2009). Physiologic effects of electroacupuncture combined with intramuscular administration of xylazine to provide analgesia in goats. Am. J. Vet. Res..

[41] Lau W.K., Lau Y.M., Zhang H.Q., Wong S.C., Bian Z.X. (2010). Electroacupuncture versus celecoxib for neuropathic pain in rat SNL model. Neuroscience.

[42] Cheng L.-L., Ding M.-X., Xiong C., Zhou M.-Y., Qiu Z.-Y., Wang Q. (2012). Effects of Electroacupuncture of Different Frequencies on the Release Profile of Endogenous Opioid Peptides in the Central Nerve System of Goats. Evid.-Based Complementary Altern. Med..

[43] Taguchi R., Taguchi T., Kitakoji H. (2010). Involvement of peripheral opioid receptors in electroacupuncture analgesia for carrageenan-induced hyperalgesia. Brain Res..

[44] Cheng L.-L., Ding M.-X., Wei J., Wu Y.-Q., Qiu Z.-Y., Chen J.-G., Liu D.-M., Hu C.-M., Hu M.-L., Shah Z. (2013). Electroacupuncture-induced dynamic processes of gene expression levels of endogenous opioid Peptide precursors and opioid receptors in the CNS of goats. Evid.-Based Complementary Altern. Med. Ecam.

[45] Zhou Z.F., Du M.Y., Wu W.Y., Jiang Y., Han J.S. (1981). Effect of intracerebral microinjection of naloxone on acupuncture- and morphine-analgesia in the rabbit. Sci. Sin..

[46] Ma C., Li C.-x., Yi J.-l., Yan L.-p. (2008). Effects of electroacupuncture on glutamate and aspartic acid contents in the dorsal root ganglion and spinal cord in rats with neuropathic pain. Zhen Ci Yan Jiu = Acupunct. Res..

[47] Cui L., Ding Y., Feng Y., Chen S., Xu Y., Li M., Hu M., Qiu Z., Ding M. (2017). MiRNAs are involved in chronic electroacupuncture tolerance in the rat hypothalamus. Mol. Neurobiol..

[48] Qiu Z.Y., Ding Y., Cui L.Y., Hu M.L., Ding M.X. (2015). The Expression Patterns of c-Fos and c-Jun Induced by Different Frequencies of Electroacupuncture in the Brain. Evid.-Based Complementary Altern. Med..

[49] Goldman J.M., Murr A.S., Cooper R.L. (2007). The rodent estrous cycle: Characterization of vaginal cytology and its utility in toxicological studies. Birth Defects Res. Part B Dev. Reprod. Toxicol..

[50] Storkson R.V., Kjorsvik A., Tjolsen A., Hole K. (1996). Lumbar catheterization of the spinal subarachnoid space in the rat. J. Neurosci. Methods.

[51] Yamada M., Fujita Y., Hayano Y., Hayakawa H., Baba K., Mochizuki H., Yamashita T. (2019). Increased Expression of Fibronectin Leucine-Rich Transmembrane Protein 3 in the Dorsal Root Ganglion Induces Neuropathic Pain in Rats. J. Neurosci..

[52] Han Q., Kim Y.H., Wang X., Liu D., Zhang Z.-J., Bey A.L., Lay M., Chang W., Berta T., Zhang Y. (2016). SHANK3 Deficiency Impairs Heat Hyperalgesia and TRPV1 Signaling in Primary Sensory Neurons. Neuron.

[53] Minfeng R., Jisheng H. (1979). Rat tail flick acupuncture analgesia model. Chin. Med. J..

